# The Dissemination of Rift Valley Fever Virus to the Eye and Sensory Neurons of Zebrafish Larvae Is Stat1-Dependent

**DOI:** 10.3390/v17010087

**Published:** 2025-01-11

**Authors:** Sebastiaan ter Horst, Aleksandra Siekierska, Ann-Sofie De Meulemeester, Arno Cuvry, Laura Cools, Johan Neyts, Peter de Witte, Joana Rocha-Pereira

**Affiliations:** 1 Department of Microbiology, Immunology and Transplantation, Rega Institute, KU Leuven, Herestraat 49, 3000 Leuven, Belgium; sebastiaan.terhorst@kuleuven.be (S.t.H.); arno.cuvry@kuleuven.be (A.C.); johan.neyts@kuleuven.be (J.N.); 2 Department of Pharmaceutical and Pharmacological Sciences, KU Leuven, Herestraat 49, 3000 Leuven, Belgium; ola.siekierska@kuleuven.be (A.S.); peter.dewitte@kuleuven.be (P.d.W.); 3 VirusBank Platform, Gaston Geenslaan 3, 3001 Leuven, Belgium

**Keywords:** bunyavirus, rift valley fever virus, innate immunity, zebrafish, pathogenesis

## Abstract

The Rift Valley fever virus (RVFV) causes haemorrhagic fever, encephalitis, and permanent blindness and has been listed by the WHO as a priority pathogen. To study RVFV pathogenesis and identify small-molecule antivirals, we established a novel In Vivo model using zebrafish larvae. Pericardial injection of RVFV resulted in ~4 log_10_ viral RNA copies/larva, which was inhibited by the antiviral 2′-fluoro-2′-deoxycytidine. The optical transparency of the larvae allowed detection of RVFV_eGFP_ in the liver and sensory nervous system, including the optic tectum and retina, but not the brain or spinal cord. Thus, RVFV-induced blindness likely occurs due to direct damage to the eye and peripheral neurons, rather than the brain. Treatment with the JAK-inhibitor ruxolitinib, as well as knockout of *stat1a* but not *stat1b*, enhanced RVFV replication to ~6 log_10_ viral RNA copies/larva and ultra-bright livers, although without dissemination to sensory neurons or the eye, thereby confirming the critical role of *stat1* in RVFV pathogenesis.

## 1. Introduction

Rift Valley fever virus (RVFV) (order: *Bunyavirales*, family: *Phenuiviridae*, genus: *Phlebovirus*) is a mosquito-borne pathogen able to cause acute viral hemorrhagic disease. It is included in the WHO list of priority diseases due to its potential to cause a public health emergency and the absence of efficacious vaccines and treatments [1]. The virus, with its segmented negative single-stranded RNA genome (ssRNA), can infect both ruminants and humans and is widely endemic throughout the African continent and the Arabian Peninsula, where it has already caused substantial outbreaks. Humans become infected through mosquito bites or via contact with body fluids or tissue of infected animals. Most humans infected with RVFV present symptoms such as self-limiting mild malaise, muscle pain, and fever. However, about ~10% of individuals develop severe symptoms such as haemorrhagic fever, encephalitis, or ocular disease [2]. Case fatality in people infected with RVFV is overall ~1% but goes up to 50% for those who develop haemorrhagic fever [3]. Ocular complications such as reduced vision, photophobia, and retroorbital pain are frequent (~2–5% of RVFV infections) and may take months to resolve or result in permanent blindness [4]. While retinal hemorrhage has been observed in some patients, it is unclear whether vascular damage and bleeding are the direct cause of the patients loss of sight [5]. This type of severe clinical outcome stresses the need to develop specific antiviral treatments and vaccines against RVFV infection.

A detailed understanding of viral dissemination, pathogenesis, and host immune response to an RVFV infection is necessary for the design and development of effective countermeasures. The remaining knowledge gaps include understanding RVFV dissemination, involved cell types in primary infection, nuances in pathogenesis between different mammals, and infection-limiting or infection-enabling host factors [6]. Small animal models play a role in unravelling these details. The virus replicates in several species, including rodents, ruminants, and non-human primates; hence, these were used in a variety of RVFV-related studies. In rodents, RVFV infection is typically lethal due to the development of hepatic necrosis, haemorrhagic disease, or encephalitis [7,8,9,10,11]. While non-human primate models best match the severe disease phenotypes of human RVFV infection, they come with major practical disadvantages and ethical concerns [12].

Over the years, zebrafish have gained ground in the field of infectious diseases and particularly of human viral diseases due to their attractive features, such as small size and vertebrate nature. On top of being better compliant with the 3 R’s (replace, reduce, and refine) rule, the model offers numerous advantages. As their larvae are optically transparent, they are ideally suited for live imaging and allow follow-up of virus dissemination throughout the different organs and systems. Moreover, zebrafish are easily genetically manipulated and have rapid development, thus resulting in a relatively fast generation of transgenic lines bearing gene knock-ins, knockouts, and/or expressing fluorophores under tissue-specific promotors. Zebrafish models to study human viral infections have been established for human herpes simplex 1 virus, influenza virus, chikungunya virus, and sindbis virus [13,14,15,16]. More recently, our team developed a zebrafish larvae model for human norovirus infections, which constituted a breakthrough given the decades-long difficulties to cultivate this important enteric pathogen [17]. Additionally, attempts have been made to infect zebrafish larvae with SARS-CoV-2, but only limited viral replication was observed in a subset of animals when infected through the swim bladder [18]. However, it was suggested that transgenic zebrafish stably expressing human ACE2 and/or transmembrane serine protease (TMPRSS2) have the potential to effectively host SARS-CoV-2 replication.

An important feature of zebrafish larvae is that they lack a mature adaptive immune system in the first 4–6 weeks post fertilization [19]. However, the innate immune system is in place from the earliest days post fertilization (dpf), with functional macrophages and neutrophils present from 30 h post fertilization, hence providing the unique possibility to dissect in detail the virus-related innate immune response separately [20]. To that end, transgenic lines bearing fluorescent neutrophils and macrophages are readily available, and the main functions and signalling pathways are conserved with the innate immune system of humans [21]. Among those, the interferon (IFN) system is a major driving force, since the ability of an individual mammalian cell to respond to a viral infection is based on the signalling through type I IFNs [22]. In zebrafish, four types of type I *ifn* are distinguished; *ifn*ϕ1-4. Many of the *ifn*-stimulated genes (ISGs) produce functional and structural orthologues to human ISGs [23]. In zebrafish, the innate immune response against ssRNA viruses is initiated upon detection of cytoplasmic viral elements by retinoic acid-inducible gene I-like receptors (RNRs) and nucleotide oligomerization domain-like receptors NLRs. ssRNA within endosomes is detected by toll-like receptors (TLRs) [24]. Subsequently, downstream transcription factor IRF-7 stimulates the production of *ifn*ϕ, thereby activating ISGs and the production of antiviral factors, such as MxA, ISG-15, and RSAD2 (also known as viperin) in neighbouring cells. One essential transducer element in the human antiviral response is STAT1, which is part of the JAK/STAT signalling pathway. In zebrafish, two paralogues are present, *stat1a* and *stat1b*, of which *stat1a* was shown to share an ancestral virus-related cytokine-signalling mechanism similar to humans [25,26]. The zebrafish *stat1a* and *stat1b* genes also show differences in expression patterns, tissue specificity, and functionality. *Stat1a* exhibits a broad expression pattern at low levels across the entire larvae, consistent with its role as a virus-related gene essential in various cell types. In contrast, *stat1b* is expressed early and abundantly in the hematopoietic tissue of zebrafish embryos and plays a key role in zebrafish haematopoiesis [26]. Interestingly, *stat1b* is strongly induced by type I *ifn*, hinting at a relationship with viral infections as well [23].

This study aimed to explore the zebrafish model to study RVFV replication, dissemination, and host innate immune response. We demonstrate that RVFV is able to replicate to high titers in zebrafish larvae and triggered a virus-specific innate immune response. Moreover, we reveal the virus tissue tropism in a whole-body organism for the first time, which largely recapitulates what is found in the human host and can be inhibited by a specific antiviral. Additionally, *stat1a* and *stat1b* knock-out zebrafish were created to investigate the role of the JAK/STAT pathway in RVFV infection and pathogenesis.

## 2. Materials and Methods

### 2.1. Ethics Statement

All animal experiments were approved and performed according to the rules and regulations of the Ethical Committee of KU Leuven (P164/2020), in compliance with the regulations of the European Union concerning the welfare of laboratory animals as declared in Directive 2010/63/EU.

### 2.2. Zebrafish Husbandry and Transgenic Lines

Routine zebrafish (*Danio rerio*) maintenance was performed under standard aquaculture conditions at 28.5 °C and a 14 h light/10 h dark cycle. Fertilized eggs were collected via natural spawning. Embryos were maintained in Danieau’s solution (1.5 mM HEPES, 17.4 mM NaCl, 0.21 mM KCl, 0.12 mM MgSO_4_, and 0.18 mM Ca(NO_3_)_2_, and 0.6 μM methylene blue) under the same conditions as adults. Wildtype AB zebrafish were obtained from ZIRC (Eugene, OR, USA). *Tg (fabp10a:DsRed; nacre)* was created as part of earlier studies in the laboratory of Peter de Witte. The *stat1a*^−/−^ and *stat1b*^−/−^ transgenic lines were generated as described below.

### 2.3. Cells and Viruses

Vero E6 cells (ATCC^®^ CRL-1586™) were cultured in Dulbecco’s Modified Eagle Medium (DMEM) (Gibco, Thermo Fisher Scientific Inc., Waltham, MA, USA) supplemented with 10% fetal bovine serum (FBS) (Gibco), 0.075 g/L sodium bicarbonate (Gibco), and 1% penicillin-streptomycin (10,000 U/mL, Gibco). End-point titrations were performed using medium containing 2% FBS. Prof. J. Kortekaas at Wageningen University and Research kindly provided the RVFV_35/74_ strain and its reporter clone RVFV_eGFP_, which expresses enhanced green fluorescent protein (eGFP) from the S-segment by replacing the NSs protein locus [27]. Virus stocks were expanded using Vero E6 cells, after which the titre (as 50% cell culture infective dose: CCID_50_) of the newly grown stocks was determined by endpoint titration on Vero E6 cells. UV-inactivated stocks were generated by distributing live RVFV stock over a 96-well plate as 100 µL/well and placing them in a closed flow cabinet with its UV disinfection cycle on for 20 min. Afterwards the UV-irradiated stocks were pooled back together into a fresh container. This, and all subsequent experiments using live virus, were performed in a BSL-3 facility.

### 2.4. Generation of the stat1a and stat1b CRISPR Knockout Zebrafish Lines

The *stat1a*^−/−^ and *stat1b*^−/−^ knockout lines were generated using the CRISPR/Cas9 technique following a modified protocol of Vejnar et al. [28]. Single guide RNAs (sgRNAs) were designed using the online tool CRISPRscan [29] targeting exon 8 of *stat1a* gene (ENSDART00000005720.6; 5′-GGCCGAGGTGTTGAACCTGG-3′) and exon 8 of *stat1b* gene (ENSDART00000000280.11; 5′-GGTCTCCAGGTTCACGGTGG-3′). The sgRNA DNA templates were synthesized via fill-in PCR using KAPA HiFi DNA Polymerase (Roche, Basel, Switzerland) and sgRNA-specific and universal primers (Table 1) and subsequently purified with the QIAquick PCR Purification Kit (Qiagen, Hongkong, China). sgRNAs mRNA was transcribed using AmpliScribe-T7 Flash Transcription kit (Epicentre, Madison, WI, USA), DNase-treated, and precipitated with sodium acetate/ethanol. To optimize the Cas9 expression and its nuclear targeting in zebrafish, we used a zebrafish codon-optimized version of *S. pyogenes* Cas9 (pT3TSnCas9n, Plasmid #46757, Addgene, Watertown, MA, USA). The plasmid was linearized with XbaI restriction enzyme and Cas9 in vitro transcription was performed using mMESSAGE mMACHINE™ T3 Transcription Kit (Invitrogen, Waltham, MA, USA). IVT sgRNAs were treated with DNase and precipitated with lithium chloride/ethanol.

Single-cell-stage fertilized wildtype embryos of the AB line were injected with a mix of 150 pg Cas9 mRNA and 100 pg *stat1a* or *stat1b* sgRNA and (in 1 nL volume) using a Femtojet 4i pressure microinjector (Eppendorf, Hamburg, Germany) and a M3301R Manual Micromanipulator (WPI, Worcester, MA, USA). After the initial injections, the efficiency of CRISPR knockout was verified using high-resolution melting (HRM) analysis with gene-specific primers. The remaining sgRNA/Cas9-injected embryos were raised until adulthood, outcrossed with WT adults of nacre background, and screened for indels by Sanger sequencing. F0 founders for *stat1a* and *stat1b* KO lines with germline transmission and a high rate of indel mutations were selected to establish the knockout line. F1-generation embryos of F0 founders were raised to adulthood, fin clipped, and Sanger sequenced using gene-specific primers. Individuals carrying heterozygous and homozygous 10-nucleotide deletion and 8-nucleotide insertion in *stat1a* and *stat1b* gene, respectively, were identified. All experiments were performed on the F2 embryos/larvae coming from homozygous knockout incrossing.

### 2.5. RNA Extraction and RT-qPCR Analysis for CRISPR Confirmation

Total RNA of 10 pooled zebrafish larvae was extracted using TRIzol reagent (Invitrogen), followed by phenol-chloroform extraction, isopropanol precipitation, and ethanol washes. The resulting RNA pellet was air-dried, dissolved in nuclease-free water (Thermo Scientific, Waltham, MA, USA), and subsequently treated with RNase-free DNase (Roche). Then, 1 µg of total RNA was reverse transcribed with the High-Capacity cDNA Reverse Transcription kit (Applied Biosystems, Waltham, MA, USA) according to the manufacturer’s protocol. Next, the generated cDNA was diluted (1:20) and amplified using *stat1a*- or *stat1b*-specific primers and 2× SsoAdvanced Universal SYBR Green Supermix (Bio-Rad, Hongkong, China) in Hardshell^®^ Low Profile Thin-wall 96-well skirted PCR plates (Bio-Rad) on a CFX96 touch RT-PCR detection system (Bio-Rad) under cycling conditions according to the manufacturer’s protocol. The relative expression levels were quantified using the comparative Ct method (ΔΔCt) with CFX Maestro 2.3 software (Bio-Rad) [30]. Transcripts were normalized against the *tuba1a* reference gene using specific primers.

### 2.6. High Resolution Melting (HRM) Analysis for Genotyping

To extract genomic DNA, a fin clip of a zebrafish was placed in 20 µL of 50 mM NaOH and heated at 95 °C for 10 min, followed by neutralization using 100 mM Tris HCl (pH 8.0) (1/10 volume). Lysed samples were genotyped by performing a PCR reaction with Precision Melt Supermix for HRM analysis (Bio-Rad #172-5112) and *stat1a-* or *stat1b*-specific primers (Table 1) in a CFX96 touch RT-PCR detection system (Bio-Rad) using Hardshell^®^ Low Profile Thin-wall 96-well skirted PCR plates (Bio-Rad). Curves were analyzed using the Precision Melt Analysis™ v1.2 Software (Bio-Rad). Genotypes/sequences of the individual larvae clustering together were confirmed with Sanger sequencing and were analyzed using SeqMan 12.2 software (DNASTAR Lasergene).

### 2.7. RVFV Injection in Zebrafish Larvae

At 3 dpf, zebrafish larvae were anesthetized by immersion in 0.4 mg/mL tricaine in Danieau’s solution. While sedated, the larvae were infected with RVFV_35/74_ or RVFV_eGFP_ in 1× PBS (in 2 nL) through microinjection into the pericardial cavity. Inoculated larvae were kept in Danieau’s solution under standard aquaculture conditions with up to 15 larvae per well of a 6-well plate up to 6 days post infection (dpi) and daily inspected under a stereomicroscope for clinical signs of infection (e.g., posture, development, swimming behaviour, or signs of edema).

### 2.8. Antiviral Treatment

2′-Fluoro-2′-deoxycytidine (2′-FdC) (Biosynth-Carbosynth, Compton, UK) was dissolved in 100% DMSO (VWR Chemicals, Philadelphia, PA, USA). The maximum non-toxic concentration of 2′-FdC in the zebrafish larvae was determined and selected according to the protocol described by Van Dycke et al. [31]. Antiviral treatment of the RVFV-injected larvae occurred immediately after infection through immersion in 2′-FdC solution, with final concentrations of 1000 µM and 10 mM.

### 2.9. Tissue Homogenization and Viral End-Point Titrations

Every dpi, ten zebrafish larvae per sample were collected and pooled together in Precellys^®^ tubes and euthanized using an overdose of tricaine. The larvae were homogenized in 300 µL DMEM in a tissue homogenizer (Precellys 24, Bertin Technologies, Montigny-le-Bretonneux, France) using 1 cycle of 5 s at 6300 rpm and centrifuged (9000 rcf, 5 min) to pellet any debris. Infectious RVFV particles in the supernatants were quantified by end-point titrations on confluent Vero E6 cells in medium containing 2% FBS in 96-well plates. Viral titres were calculated with the Reed and Muench method using the Lindenbach calculator and were expressed as TCID_50_ per larvae [32].

### 2.10. RNA Extraction and Viral RNA Quantification

Every dpi, ten zebrafish larvae per sample were collected in Precellys^®^ tubes and euthanized using an overdose of tricaine. The larvae were homogenized in 350 µL RLT-lysis buffer (Qiagen) in a tissue homogenizer (Precellys 24, Bertin Technologies) using 1 cycle of 5 s at 6300 rpm. Total RNA was extracted using the RNeasy^®^ Mini kit (Qiagen), according to the manufacturer’s protocol.

Viral RNA was detected by RT-qPCR iTaq™ Universal SYBR^®^ Green One-Step Kit (Bio-Rad, Hongkong, China) using RVFV-specific primes (Table 1). Cycling conditions: reverse transcription at 50 °C for 10 min, initial denaturation at 95 °C for 3 min, followed by 40 cycles of amplification (95 °C for 15 s, 60 °C for 30 s) (Roche LightCycler 96, Roche Diagnostics, Basel, Switzerland). For absolute quantification, standard curves were generated using 10-fold dilutions of DNA template of known concentrations.

**Table 1:** List of primers.

Primer		Sequence (5′-3′)	Ref.
*stat1a* sgRNA (fill-in PCR)	-	TAATACGACTCACTATAGGCCGAGGTGTTGAACCTGGGTTTTAGAGCTAGAA	-
*stat1b* sgRNA (fill-in PCR)	-	TAATACGACTCACTATAGGTCTCCAGGTTCACGGTGGGTTTTAGAGCTAGAA	-
Universal (fill-in PCR)	-	AAAAGCACCGACTCGGTGCCACTTTTTCAAGTTGATAACGGACTAGCCTTATTTTAACTTGCTATTTCTAGCTCTAAAAC	-
*stat1a* (HRM)	Forward	GGCGATTAGTCAGATGTCCG	-
	Reverse	ATCAGCTCACAGATCACCGG	
*stat1b* (HRM)	Forward	AGCGTGACTTGTTCTCCAGG	-
	Reverse	GCTGGCCCCTTCCTAGATTT	
*stat1a* (CRISPR confirm)	Forward	GTCAGAGAGTCCAACACCGA	-
	Reverse	TTCACCCTTGCGTCCATTTC	
*stat1b* (CRISPR confirm)	Forward	TGTGCAGGAAATGGAAAAGCA	-
	Reverse	TCTCTTTTGGCATCGGGTCA	
*tuba1a*	Forward	AGGTCTCCACAGCAGTAGTAGACC	-
	Reverse	GTCCACCATGAAGGCACAGTCG	
RVFV	Forward	AAAGGAACAATGGACTCTGGTCA	[32]
	Reverse	CACTTCTTRCTACCATGTCCTCCAAT	
*18s*	Forward	CGGAGGTTCGAAGACGATCA	[33]
	Reverse	TCGCTAGTTGGCATCGTTTATG	
*β* *-actin*	Forward	ATGGATGAGGAAATCGCTG	[34]
	Reverse	ATGCCAACCATCACTCCCTG	
*stat1a*	Forward	AGTCGCAGCAATGACTCAGTG	-
	Reverse	CCCAGTCGTGGCTTTCT	
*stat1b*	Forward	GGTGACTCCATGCAGGGG	-
	Reverse	CGTAATCATCTTGCATATCCTCC	
*ef1* *α*	Forward	GCTGATCGTTGGAGTCAACA	[35]
	Reverse	ACAGACTTGACCTCAGTGGT	
*irf7*	Forward	TCTGCATGCAGTTTCCCAGT	[15]
	Reverse	TGGTCCACTGTAGTGTGTGA	
*ifnϕ1*	Forward	TGAGAACTCAAATGTGGACCT	[15]
	Reverse	GTCCTCCACCTTTGACTTGT	
*mxa*	Forward	ATAGGAGACCAAAGCTCGGGAAAG	[34]
	Reverse	ATTCTCCCATGCCACCTATCTTGG	
*isg15*	Forward	AACTCGGTGACGATGCAGC	[36]
	Reverse	TGGGCACGTTGAAGTACTGA	
*rsad2*	Forward	GCTGAAAGAAGCAGGAATGG	[35]
	Reverse	AAACACTGGAAGACCTTCCAA	
*scl*	Forward	TCCCAGAGACCCGCTGAGCG	[37]
	Reverse	CAGGAGGGTGTGTTGGGATG	
*gata1*	Forward	GAATGCAGCTTCAGAGGTTTATCC	[26]
	Reverse	TGGGTTCAGAGAATACGCTCCTA	
*spi1*	Forward	TCAAATGAAAAGCAGCGTCATATTC	[38]
	Reverse	CCATAGCACATCATGAAAGTTCAC	
*mpx*	Forward	CCAAACCTCAGGGATGTTCTTG	[26]
	Reverse	CCCAAACTACGAGTCCCTATGC	
*mpeg*	Forward	ATCAGTGTCTGCAACCTGCAT	[39]
	Reverse	TTGCCACTTCTGCAGAGTGAT	

### 2.11. Quantification of Expression of Innate Immune-Related Gene Expression

cDNA was generated using the ImProm-II Reverse Transcription System (Promega, Madison, WI, USA) by adding 1 μL of random hexamers to a total of 1 μg of extracted total RNA and incubating at 70 °C for 5 min, followed by 5 min at 4 °C. A mix of 8 μL of Improm II 5× reaction buffer, 6 mM MgCl_2_, 0.5 mM deoxynucleoside triphosphate, 40 units of RNase inhibitor, and 1 μL of Improm II reverse transcriptase was mixed and incubated at 25 °C for 5 min, 37 °C for 1 h, and 72 °C for 15 min. qPCR was performed using SsoAdvanced™ Universal SYBR^®^ Green Supermix and 500 nM of gene-specific forward and reverse primers (Table 1) with cycling conditions of 95 °C for 3 min followed by 40 cycles of 95 °C for 15 s, 55 °C for 30 s, and 72 °C for 30 s (Roche LightCycler 96, Roche Diagnostics). Data were normalized against the housekeeping genes (*18 s*, *β-actin*, and *ef1α*) and compared with PBS-injected zebrafish larvae to determine the fold induction of the expression, according to the 2^ΔΔCt^ method [30].

### 2.12. Whole Mount Immunochemistry and Imaging

Zebrafish larvae from 0 to 6 dpi were anesthetized in 0.4 mg/mL tricaine in Danieau’s solution and fixed in 3.8% formaldehyde in 1× PBS. After fixation, three wash steps were performed using 1× PBS supplemented with 0.1% Tween-20 (1× PBST). From this point, the larvae were exported out of the BSL-3 facility for further processing and analyses in laboratories of lower BSL. Permeabilization was achieved starting with a wash step of 30 min in distilled H_2_O, followed by 20 min incubation in 100% acetone at −18 °C, after which the larvae were washed three times with HBSS (Gibco), followed by immersion in collagenase (1 mg/mL in HBSS) for 60 min. After three washing steps using 1× PBST and 1% DMSO (1× PBSDT), the larvae were blocked for 1 h in a blocking solution (10% sheep serum in 1× PBSDT) and incubated overnight at 4 °C in the blocking solution containing primary antibody (Table 2). Next, the larvae were washed (4× with 1× PBSDT), with the final washing step of at least 2 h at room temperature. An additional blocking step was performed for 1 h in the blocking solution, and afterwards the larvae were incubated overnight at 4 °C in the blocking solution containing secondary antibody (Table 2). The next day, the larvae were washed (3×, 1× PBST) and incubated for 30 min in 2 µg/mL Hoechst 33342 (Invitrogen) in 1× PBT at room temperature. After the incubation, larvae were washed six times in 1× PBST over the course of 3 h. RVFV infection scoring and image acquisition were performed with a Leica DMI8 microscope, using 10× dry objective (NA 0.32) or 20× dry objective (NA 0.40). Image processing was performed in LAS X software, version 3.7.4 (i.e., panel merging, optimal projections, and 3D deconvolution, using default set parameters).

### 2.13. Statistical Analysis

GraphPad Prism (GraphPad v9.4 Software, Inc., New York, NY, USA) was used to perform statistical analysis. To evaluate differences between means, two-tailed unpaired Student *t*-test was used. To determine normal distributions, Shapiro–Wilk tests were performed. Nonparametric data were evaluated using the Kruskal–Wallis test. Survival studies were analyzed using Log-rank (Mantel–Cox) test. Values *p* < 0.05 are considered statistically significant (* *p* < 0.05; ** *p* < 0.01; *** *p* < 0.001; **** *p* < 0.0001; ns, not significant).

## 3. Results

### 3.1. RVFV Replicates in Zebrafish Larvae and Induces an Innate Immune Response

**Figure 1:**
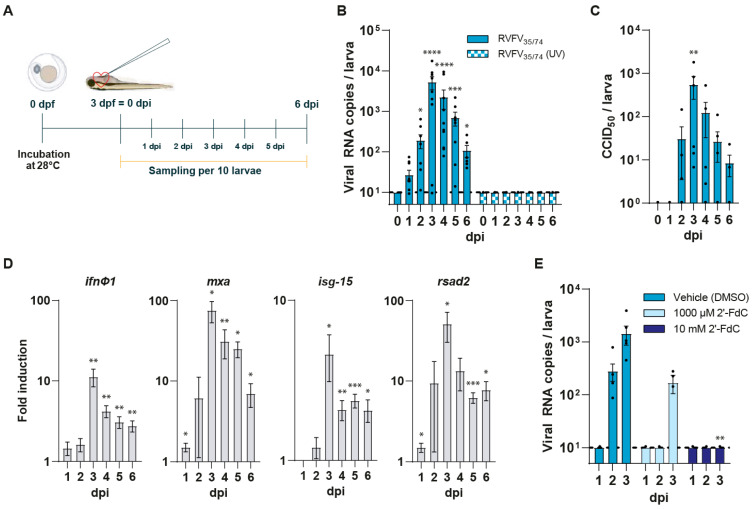
RVFV infection in zebrafish larvae resulting in viral replication and innate immune response. (**A**) Schematic representation of the experimental timeline, conditions, and injection site. Drawings acquired through BioRender. (**B**) Viral RNA copies of RVFV_35/74_ per zebrafish larva. Mean ± standard error of the mean (s.e.m.) of 5–8 pools of 10 larvae from 8 independent experiments. The dotted line shows limit of quantification (LOQ). (**C**) Titres of RVFV_35/74_ infectious viral particles in zebrafish larvae. Mean ± s.e.m. of 6 pools of 10 larvae from 6 independent experiments. (**D**) Relative fold induction of *inf*ϕ1, mxa, isg-15, and rsad2 in RVFV_35/74_ infected zebrafish larvae compared to PBS-injected zebrafish larvae. Results determined by RT-qPCR and normalized to 18 s, ef1α, and β-actin at 0 dpi. Mean ± s.e.m. of 8 pools of 10 larvae from 8 independent experiments. (**E**) Viral RNA copies of RVFV_35/74_ per zebrafish larva after immersion in Danieau’s solution containing the vehicle, 1000 µM, or 10 mM 2′-FdC at 0 dpi. Mean ± s.e.m. of 5 pools of 10 larvae from 5 independent experiments. The dotted line shows LOQ. dpf = days post fertilization; dpi = days post infection; RVFV = Rift Valley fever virus; 2′-FdC = 2′-Fluoro-2′-deoxycytidine; * *p* < 0.05; ** *p* < 0.01; *** *p* < 0.001; **** *p* < 0.0001; ns = not significant.

To establish a zebrafish larvae infection model for RVFV, we injected ~1 CCID_50_ of RVFV_35/74_ (in a 2 nL volume) or a UV-irradiated sample of the same virus in the pericardial cavity of 3 dpf larvae (Figure 1A). The 1 CCID_50_ infection dose was chosen because of robust replication yields and undetectable background signal from the inoculum. (Figure S1) This site of injection was selected as it quickly introduced the virus to the bloodstream and towards relevant tissues. RVFV_35/74_ viral RNA was detected from 1 dpi onwards, reaching a peak of ~4 log_10_ RNA copies per larva at 3 dpi (Figure 1B). In the following days, viral RNA load slowly decreased to ~2 log_10_ RNA copies per larva at 6 dpi. When the larvae were injected with the UV-irradiated inoculum, no increase in viral RNA, hence no viral replication, was observed (Figure 1B). Additionally, infectious virus titres were determined by endpoint titration to confirm the presence of infectious virus progeny. The infectious virus titres coincided with the detected RNA levels, with a peak observed at 3 dpi, confirming that viral RNA detection correlates with the presence of infectious virions (Figure 1C). No signs of disease (emaciation, skeletal deformation, edema, or hemorrhages) were detected in the RVFV-injected larvae at any time during the experiment, and no difference in mortality was observed between the larvae inoculated with the virus or PBS as a control (Figure S2A).

To demonstrate that zebrafish mount an innate immune response upon infection with RVFV, we quantified the mRNA expression levels of innate immunity-related genes. Upon infection with RVFV_35/74,_, the expression of interferon-ϕ1 (*ifn*ϕ*1*) and downstream ISGs *mxa*, *isg-15*, and *rsad2* were increased, with maximum levels coinciding with the peak of virus replication at 3 dpi (Figure 1D).

To validate our model for antiviral studies and generate additional evidence that RVFV can effectively replicate in zebrafish; we treated RVFV_35/74_-infected larvae with 2′-Fluoro-2′-deoxycytidine (2′-FdC), a nucleoside polymerase inhibitor with a proven in vitro and in vivo antiviral effect against RVFV [40]. Immediately after virus infection (0 dpi), the larvae were exposed to 10 mM 2′-FdC, after which no viral replication was detected for the first 3 dpi. When a 10-fold lower concentration of 2′-FdC was used, no viral RNA was detected at 1 and 2 dpi, while at 3 dpi viral RNA levels were detectable but still lower than those found in DMSO-treated controls (Figure 1E). Toxicity scoring of 2′-FdC in the zebrafish larvae was performed following the protocol described by Van Dycke et al., with the drug being not toxic at the selected concentrations [31].

Taken together, we demonstrated that zebrafish larvae are permissive to RVFV infection and viral replication, which induced a virus-induced innate immune response. Moreover, treatment with an antiviral compound completely prevented RVFV replication, demonstrating the utility of the zebrafish model for testing and identification of novel small molecule inhibitors of RVFV replication.

### 3.2. RVFV Has a Tropism for the Liver and Sensory Nervous System in Zebrafish Larvae

**Figure 2:**
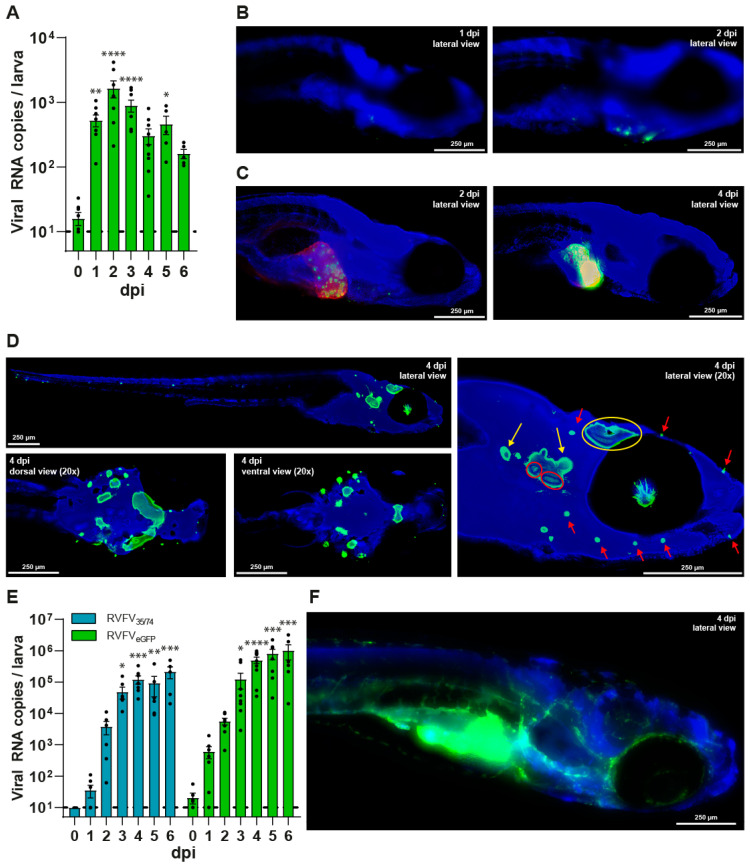
Viral replication and dissemination of RVFV_eGFP_ in zebrafish larvae, with infection of the liver, sensory nervous system and vascular system. (**A**) Viral RNA copies of RVFV_eGFP_ per zebrafish larva. Mean ± s.e.m. of 6–8 pools of 10 larvae from 8 independent experiments. (**B**–**D**,**F**) Whole mount immunohistochemistry fluorescence images of RVFV_eGFP_-infected zebrafish larvae at 10× magnification, using anti-GFP primary antibody and Hoechst 33342. (**B**) Wildtype zebrafish larva at 1 and 2 dpi showing progressing RVFV infection around the site of injection. (**C**) *Tg (fabp10a:DsRed; nacre)* zebrafish larvae demonstrating infection in the liver using anti-RFP primary antibody. (**D**) Wildtype zebrafish larva showing infection of the retina, optic tectum (yellow circle), the anterior and posterior macula (red circle), the ganglia (yellow arrows), and neuromasts (red arrows) of the lateral line. dpi = days post infection. (**E**) Viral RNA copies of RVFV_35/74_ and RVFV_eGFP_ per zebrafish larva immersed in Danieau’s solution supplemented with ruxolitinib (25 µM) from 0 dpi. Inoculum: ~1 CCID_50_ of RVFV_35/74_ or ~15 CCID_50_ of RVFV_eGFP_. Mean ± s.e.m. of 8 pools of 10 larvae from 8 independent experiments. (**F**) Wildtype zebrafish larva at 6 dpi, showing extensive dissemination of RVFV_eGFP_ infection after immersion in Danieau’s solution supplemented with ruxolitinib (25 µM) from 0 dpi. Image has not been 3D-deconvoluted. dpi = days post infection; RVFV = Rift Valley fever virus; * *p* < 0.05; ** *p* < 0.01; *** *p* < 0.001; **** *p* < 0.0001; ns = not significant.

To investigate the tropism of RVFV upon infection in zebrafish larvae, we used an eGFP fluorescently labelled RVFV_35/74_ strain, hereafter named RVFV_eGFP_. This strain expresses eGFP from the S-segment by replacing the NSs protein locus [27]. Infection with ~15 CCID_50_ of RVFV_eGFP_ in the pericardial cavity of the larvae yielded similar replication kinetics, but with an earlier peak of replication of ~3 log_10_ RNA copies per larva at 2 dpi (Figure 2A). Of note, a 15× higher inoculum was used in comparison to the wildtype strain to achieve a similar infection yield and minimal background signal (Figure S1). In line with what was previously reported, the reporter strain showed attenuation due to the lack of NSs protein expression [41]. However, as in experiments with the wildtype virus, there was no difference in mortality between RVFV_eGFP_-infected larvae and PBS-injected controls throughout the experiments (Figure S2A). By means of whole mount immunohistochemistry and fluorescence microscopy, we were able to score and image RVFV_eGFP_-infected zebrafish larvae starting from 1 dpi (Figure 2B and Table S1). After infection, on average 48 out of 60 larvae (*n* = 3) showed a RVFV_eGFP_-positive signal around the site of injection at 1 or 2 dpi. Larvae harvested after 2 dpi developed an infection of the liver in a spot-like pattern during the early days of the infection, which became more extensive at 3–6 dpi resulting in a completely infected liver. Infection of the liver was confirmed by inoculation of RVFV_eGFP_ of a transgenic zebrafish line, *Tg (fabp10a:DsRed; nacre)*, expressing DsRed fluorophore in the hepatocytes. Upon infection, we observed an overlap of the red fluorescent signal from the hepatocytes and the eGFP translated from the viral genome (Figure 2C), reiterating that the liver is the main target organ for RVFV replication in zebrafish, akin to humans. In two out of six independent experiments, nearly all larvae showed infection of their sensory nervous system, including the optic tectum, the anterior and posterior macula (both part of the inner ear), the lateral line including the related ganglia, and the retina (Figure 2D) (Video S1 and Table S2). The typical pattern of the neuromasts belonging to the lateral line around the head and along the body of the larvae was clearly distinguishable. Out of these 60 larvae, we found two larvae that did show infection of the optical tectum and the most proximal neuromasts, but without having spread to the more distal parts of the lateral line (Figure S3). Remarkably, none of the larvae showed an overlapping infection pattern including both the liver and the sensory nervous system. To confirm the green, fluorescent signal was infection-specific, a total of 180 uninfected (PBS-injected) larvae from the same batch of eggs served as a negative control and were also stained using the anti-GFP antibody. These larvae did not show any GFP signal (Figure S4 and Table S2).

To confirm the role of the JAK/STAT signalling pathway in RVFV infection, we exacerbated the viral infection by introducing the JAK signalling inhibitor ruxolitinib to the swimming water of the zebrafish larvae. The maximum tolerated concentration of ruxolitinib in the zebrafish larvae was determined following the protocol described by Van Dycke et al. and a non-toxic concentration was selected [31]. Viral RNA levels were increased upon infection with both the RVFV_35/74_ strain and the RVFV_eGFP_, reaching ~5 log_10_ RNA copies/larva at 3 dpi (Figure 2E). Beyond that timepoint, viral RNA levels continued to increase to values close to ~6 log_10_ RNA copies/larva at 6 dpi, which contrasts with the ~2 log_10_ RNA copies/larva found in the above shown when larvae were left untreated. This observation supports a key role for the JAK/STAT pathway in the host response to the infection in zebrafish larvae. Imaging of RVFV_eGFP_-infected larvae at 6 dpi showed extensive dissemination of the virus. Their abdominal area was bright green as the virus had infected the liver, large portions of the abdominal cavity and gastrointestinal tract, and their entire vascular system (Figure 2F). While eGFP signal was detected throughout the head and the area surrounding the eye, the pattern of observed fluorescence indicates that it was not the neurons that were infected, but rather the vascular system. In fact, none of the larvae showed any signs of infection in the sensory nervous system, lateral line, or retina. Moreover, despite the more extensive replication and dissemination of the virus in larvae treated with the JAK inhibitor, their survival rates were not affected (Figure S2B).

### 3.3. Inhibiting the JAK/STAT Pathway Exacerbates RVFV Replication and Dissemination in Zebrafish Larvae

**Figure 3:**
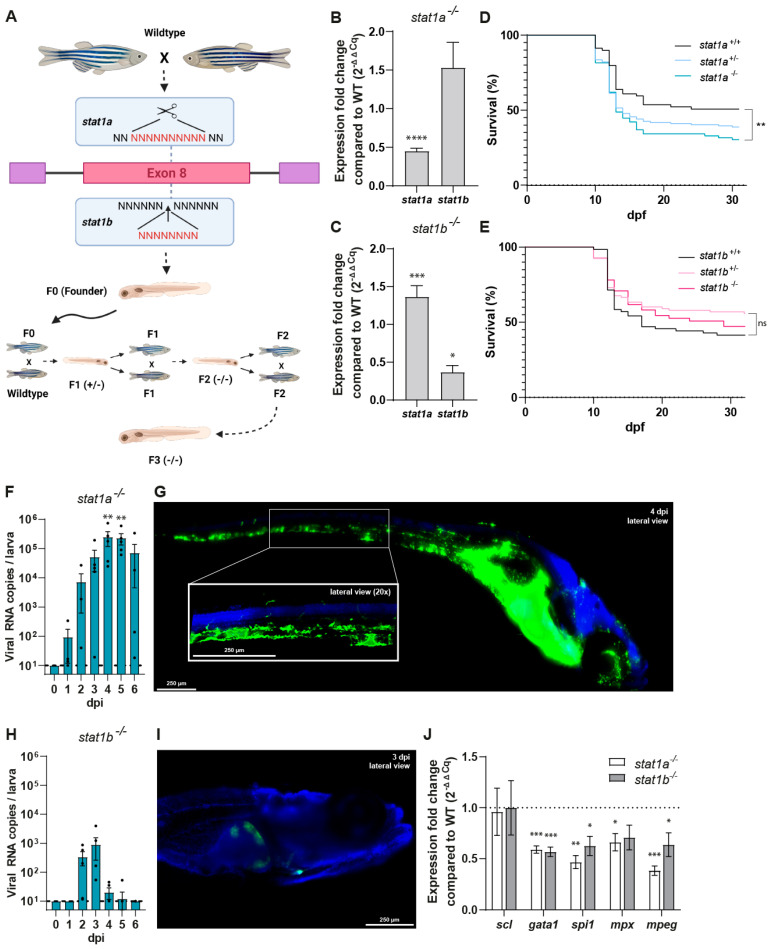
The effect of stat1a and stat1b knockout on RVFV viral replication, dissemination, and innate immune response. (**A**) Schematic representation of the creation of stat1a^−/−^ and stat1b^−/−^ larvae. Created in BioRender. (**B**) The relative mRNA expression fold change in stat1a and stat1b in stat1a^−/−^ zebrafish larvae. (**C**) The relative mRNA expression fold change in stat1a and stat1b in stat1b^−/−^ zebrafish larvae. (**D**) Survival curves of stat1a^+/+^ (*n* = 69), stat1a^+/−^ (*n* = 129), and stat1a^−/−^ (*n* = 74) larvae of the F2 generation. (**E**) Survival curves of stat1b^+/+^ (*n* = 71), stat1b^+/−^ (*n* = 92), and stat1b^−/−^ (*n* = 55) larvae of the F2 generation. (**F**) Viral RNA copies of RVFV_35/74_ per stat1a^−/−^ zebrafish larva. Inoculum: ~1 CCID_50_ of RVFV_35/74_. Mean ± s.e.m. of 5–6 pools of 10 larvae from 6 independent experiments. (**H**) Viral RNA copies of RVFV_35/74_ per stat1b^−/−^ zebrafish larva. Inoculum: ~1 CCID_50_ of RVFV_35/74_. Mean ± s.e.m. of 6 pools of 10 larvae from 6 independent experiments. (**G**,**I**) Whole mount immunohistochemistry fluorescence images of RVFV_eGFP_ infected stat1a^−/−^ (**G**) and stat1b^−/−^ (**I**) zebrafish larvae at 10× magnification, using anti-GFP primary antibody (green) and Hoechst 33342 (blue). Inoculum: ~15 CCID_50_ of RVFV_eGFP_. Stat1a^−/−^ zebrafish larva at 4 dpi, showing extensive dissemination of RVFV_eGFP_ infection. Stat1b^−/−^ zebrafish larva at 3 dpi, showing limited dissemination of RVFV_eGFP_ infection. (**J**) Fold induction of gene expression relative to wildtype larvae of hematopoietic markers in stat1a^−/−^ and stat1b^−/−^ larvae, respectively, at 6 dpf. Results determined by RT-qPCR and normalized to 18s, ef1α, and β-actin mRNA. dpi = days post infection; RVFV = Rift Valley fever virus; WT = wildtype; * *p* < 0.05; ** *p* < 0.01; *** *p* < 0.001; **** *p* < 0.0001; ns = not significant.

As *stat1* is an important signal transducer in the JAK/STAT signalling pathway, its loss of function was investigated in zebrafish. Due to genome duplication in zebrafish, two paralogues of the human *STAT1* gene exist, i.e., *stat1a* and *stat1b*, and thus two zebrafish knockouts were generated. Using CRISPR/Cas9 genome editing technology [42], we targeted the 8th exon of the zebrafish *stat1a* and *stat1b* genes, encoding a part of the ‘coiled-coil’ domain of the respective proteins. For the *stat1a* gene, a positive founder transmitting a 10-nucleotide frameshifting deletion was selected, and for the *stat1b* gene, we chose a positive founder carrying an 8-nucleotide insertion; both would result in a premature stop codon and consequently truncated proteins (Figure 3A). The genomic sequence of both *stat1a* and *stat1b* mutant larvae was confirmed by Sanger sequencing, and the selected founders were crossed to obtain the homozygous larvae in the F2 generation. These F2 homozygotes survived until adulthood, were fertile, and the F3 offspring from the homozygous parents was used for all subsequent experiments. Quantification of mRNA expression showed a significant reduction in respective *stat1* gene expression in homozygous knockout larvae (Figure 3B,C), likely resulting from nonsense-mediated mRNA decay. In the *stat1b*^−/−^ larvae, a decrease in *stat1b*^−/−^ mRNA expression was confirmed and accompanied by a remarkable increase in *stat1a* expression of ~1.5-fold. No obvious morphological defects were observed in these F3 generation *stat1a*^−/−^ and *stat1b*^−/−^ larvae. Survival studies during raising to adulthood demonstrated that *stat1a*^−/−^ larvae had a decreased survival rate, likely due to a higher susceptibility to infections, while the survival of *stat1b*^−/−^ larvae was not affected (Figure 3D,E).

Upon injection of the *stat1a*^−/−^ larvae with RVFV_35/74_, we observed a similar course of infection as for wildtype larvae treated with ruxolitinib, with viral loads above 5 log_10_ RNA copies per larva (Figure 3F). Similarly, viral RNA levels did not rapidly diminish after 3 dpi but remained stable until the end of the experiment. In addition, the dissemination pattern of RVFV_eGFP_ seems analogous to ruxolitinib-treated larvae, with a very bright signal in the liver and widespread infection of the vascular system (Figure 3G). While virus dissemination is extensive, there was no effect on the survival rates over the course of the experiment (Figure S2C). Replication of RVFV_35/74_ was impaired in *stat1b*^−/−^ larvae with only minor levels of viral RNA detected at 2, 3, and 4 dpi (Figure 3H). Regarding the dissemination of RVFV_eGFP_, no changes in the site of replication were detected; the eGFP signal was mainly found around the place of injection and in a few cases in the liver (Figure 3I). The overexpression of *stat1a* in *stat1b*^−/−^ larvae could explain, at least in part, the lowered RVFV replication. In addition, *stat1b* was described to play a role in haematopoiesis, promoting myeloid development at the expense of erythroid development [26]. We hypothesize that the absence of *stat1b* conditioned the number of monocyte/macrophages present in the larvae, which affected the ability of the virus to infect and propagate within the larvae. In fact, it was confirmed that cells of the myeloid lineage are a host for RVFV and a determining factor in viral dissemination [43]. Hence, we investigated potential differences in the leukocyte population in both *stat1a*^−/−^ and *stat1b*^−/−^ larvae compared to wildtype larvae at 6 dpf. We used *scl* as a marker for hematopoietic progenitor cells, *gata1* for erythrocytes, *spi1* (*pu1*) for myeloid precursors, *mpx* for heterophil granulocytes, and *mpeg* for monocytes. In line with what was suggested by Song et al. [26], we detected low levels of the myeloid lineage precursor marker *spi* and both *mpx* and *mpeg* markers (Figure 3J). However, these levels were the same in the *stat1a*^−/−^ line, where *stat1b* is abundantly present. Moreover, we detect lower levels of the erythrocyte marker *gata1* in both knockout lines compared to wildtype, which is also not in line with the expected shift of haematopoiesis towards erythropoiesis. Only the marker for hematopoietic progenitor cells, the *stat1a*^−/−^ and *stat1b*^−/−^ larvae, showed similar levels compared to wildtype larvae.

## 4. Discussion

Here, we report a new infection model for RVFV using zebrafish larvae. In this model we observed extensive viral replication and dissemination to organs compatible with what has been described in the human host. While mouse models for RVFV have already been described, zebrafish as a small vertebrate model is a worthwhile endeavour. Even though zebrafish are evolutionarily more distant from humans than rodents, they have high genetic similarity, with approximately 82% of human disease-related genes having at least one orthologue in zebrafish [44]. On top of the higher compliance with the ethical 3Rs rule (particularly when used before 120 h post fertilization), zebrafish are specifically advantageous for high biosafety level (BSL) environments due to their small size, ease of manipulation and transparency. Their small size enables the use of 96-well plates for housing and thereby generates lower volumes of biohazard infectious waste compared to rodents (cages, bedding, food, water, cadavers, etc.). Moreover, virus injection procedures do not require the use of sharps, eliminating the risk of needlestick injuries, which is particularly relevant in this high BSL setting. Additionally, zebrafish transparency facilitates the visualization of viral dissemination throughout the course of infection with a reporter virus and thus provides novel insights into RVFV infection. As possible limitations of the use of this model, we point out that the zebrafish is less widely used as a laboratory animal than rodents. The pros and cons of the species organism and its translatability are less well described, and a smaller pool of zebrafish-specific reagents is available. These aspects will become clear once the popularity of the zebrafish as a model organism continues to grow.

Over the years, zebrafish have been used for the identification of bioactive compounds, toxicity studies, and beyond, both in manual and automated settings [45,46]. Here, we studied the in vivo efficacy of the known broad-spectrum viral polymerase inhibitor, 2′-FdC, active against RVFV infection in mice [40]. The treatment proved to be efficient, yielding ~2 log_10_ reduction in virus progeny at the highest tested concentration. In this way, we showed that antiviral activity was also recapitulated in a zebrafish larval model and thus can be used for the discovery of other (novel) antiviral drugs. Other advantages of the zebrafish model include the capacity to immediately and easily monitor compound toxicity in a complete model organism and the low amounts of compound needed for drug testing [46]. Moreover, if the solubility of the compound allows, it can be simply added to the swimming water without specific formulation, being absorbed by mouth, gills, or skin (or a combination thereof). While this enables a quick testing of multiple preclinical candidates, it is limited in the fact that dose calculations cannot be immediately derived. This will require the setup of adequate drug quantification methods (e.g., Ultra performance liquid chromatography-mass spectroscopy UHPLC). Less water-soluble molecules can be injected in the pericardial cavity or caudal vein at a known dose of x mg/kg. Overall, this demonstrates that zebrafish models are simple and affordable tool organisms, which can be used early in a drug discovery campaign to generate invaluable high-quality in vivo data by using very limited resources.

Upon injection of RVFV into 3 dpf zebrafish larvae, the virus was able to replicate efficiently to high titres. Pericardial injection was chosen as the way of inoculation to directly release the virus into the bloodstream of the larva. The infection progressed rapidly with a swift rise in viral titres, akin to what is observed in studies using mice [8]. After the infection peaked at 3 dpi, we observed a gradual decrease in viral RNA and infectious particles, which can be attributed to the innate immune response mounted by the larvae. IFN plays an important role in the immunity against infections by negative-strand RNA viruses, including RVFV [47]. The involvement and close relationship between the IFN-competency of the host and its immunogenic response to RVFV infection is well established. The ability of RVFV to inhibit IFN pathway functionality correlates with increased viremia [48,49].

We also demonstrated that in zebrafish larvae *ifn*ϕ and several downstream ISGs are strongly induced early upon infection, successfully controlling the RVFV infection. The contribution of the innate immune system can be assured, as the adaptive immunity is not functional yet at this stage in larval development [19].

Using an RVFV_eGFP_, we were able to visualize the RVFV infection and dissemination throughout the body of the larvae. This reporter virus lacks the NSs gene, which is replaced by the *eGFP* gene that is expressed upon infection of a host cell. The lack of the NSs protein might have attenuated the virus, as inoculum of comparable titres to the wildtype virus did not consistently provoke an infection in the larvae. For this reason, we resorted to a ~10-fold higher inoculum in all further experiments. We believe this increase in inoculum was the reason that the virus was able to reach substantial RNA levels a day earlier when compared to our experiments using wildtype virus. We found that small spots of eGFP signal could be detected as early as 1 dpi around the site of infection in most of the larvae. The infection did not progress identically in all RVFV_eGFP_-infected larvae. At 2 dpi, an average of 48 out of 60 of the larvae showed infection around the site of injection, from where the infection spread to the liver at later dpi. This is of particular interest since RVFV is known to be hepatotropic in both human patients and mouse models [50]. In the subsequent days, an increasing number of larvae displayed infection of the liver at varying degrees of severity. Remarkably, from 2 dpi onwards we observed infection of the sensory nervous system of the larvae from two out of six independent experiments. So far, our ability to pinpoint how and where the virus transfers from the liver or vascular system into the sensory nervous system was hampered due to the limited experimental setup in the BSL-3 environment. Live imaging could help to investigate this further in the future. Since 2 out of a total of 60 larvae showed infection restricted to the optic tectum, without it having spread (yet) to the lateral line or retina, we speculate that the optic tectum might be the gateway for the virus to spread into the sensory nervous system and progressively to other sensory neuron-rich structures. The other positive larvae (28 out of 60) displayed infection throughout their broader sensory nervous system, showing fluorescent signal in all related structures, i.e., the optic tectum, the retina, the anterior and posterior macula, the ganglia, and the neuromasts of the lateral line. These structures have been shown to be connected by ensembles of neurons within the tectum that respond to sound, flow, or visual stimuli [51]. This co-localization of neural clusters might provide the virus with an excellent site from where to disseminate and travel along afferent neuronal axons, afterwards infecting the innervated structures. Interestingly, we observed the presence of the virus in a branched structure within the eye, suggesting infection and potential damage to the eye or optic nerve, which might potentially be linked to the RVFV-related blindness in humans [52,53]. A recent study using Sprague Dawley rats showed that ocular structures, including the retina and optic nerve, are permissible to RVFV infection and that even in the absence of significant viral burden, inflammation of the eyes can be observed [54]. Even though the virus is able to infect sensory nervous system-related tissues and the optic tectum, it does not seem able to further spread into other areas of the central nervous system, since we never observed infection of the brain outside of the optic tectum or spinal cord. While the maturation of the zebrafish BBB occurs between 3 and 10 dpf, expression of high-resistance tight junction proteins claudin-5 and ZO-1 is already present at 3 dpf, leading to reduced permeability of the brain microvasculature [55]. Our finding is contradictory to earlier studies in RVFV-infected rats and ferrets, where acute encephalitis and infection of the brain were observed [56,57]. However, it should be noted that in those studies the animals were infected through aerosol exposure, rather than via intravenous or subcutaneous administration, which alters the pathogenesis of RVFV [58]. A similar approach, i.e., RVFV inoculation through the olfactory system, could be used in zebrafish adults and eventually, in larvae [55].

Immersion in water with the JAK inhibitor ruxolitinib exacerbated the infection and hampered its termination due to the inhibition of the JAK/STAT pathway. A very intense fluorescent signal was detected in the liver, which interfered with the imaging of the surrounding tissue. Likewise, the complete vascular system of the larvae lit up green, including microvasculature in and around the eyes, the brain, and the muscles. Despite this massive spread in the blood vessels, there was no fluorescent signal observed within surrounding neuronal, muscular, or connective tissue. The lack of fluorescent signal in the sensory nervous system of these JAK/STAT-inhibited larvae indicates immune-dependent dissemination of the virus in larvae with a pharmacologically inhibited JAK/STAT pathway.

To further study the role of the JAK/STAT pathway and more specifically of its transducer STAT1 in RVFV infection, we generated two knockout zebrafish lines, *stat1a*^−/−^ and *stat1b*^−/−^. Knockout of the respective genes was confirmed; however, mRNAs were still detectable but at a significantly lower quantity. As expected, the *stat1a*^−/−^ larvae showed a similar infection progression as larvae treated with ruxolitinib. This confirms that *stat1a* is indeed a key factor in triggering the host innate immune response against RVFV infection. In *stat1a*^−/−^ larvae, the virus encountered minimal resistance and was able to disseminate extensively throughout the liver and vascular system. Remarkably, despite the high viral loads, none of our experiments showed infection of any component of the sensory nervous system, either when using ruxolitinib or *stat1a*^−/−^ larvae. Similar findings regarding immune-dependent pathophysiology were very recently reported, namely an unexpected lack of encephalitis in surviving IFNAR^−/−^ mice [59].

When infecting *stat1b*^−/−^ larvae, much lower viral loads were detected, with the majority of larvae showing eGFP signal only at the site of infection and a small fraction showing infection of the liver. This could be explained by the significantly increased levels of *stat1a* found in these larvae. We hypothesize that the increase of *stat1a* in *stat1b*^−/−^ larvae arises due to increased stimulation of the *stat1a* pathway caused by impaired downstream processes of stat1b (e.g., myeloid lineage development) for which the innate immunity tries to find a counterbalance. Such a genetic compensation in response to a gene knockout has already been described many times in several model organisms [60]. Since *stat1a* and *stat1b* genes are paralogues, they are thought to serve distinct functions; thus, this apparent connection was a surprising finding. The higher levels of *stat1a* in *stat1b*^−/−^ larvae might account for the lower viral load and therefore directly lessen viral dissemination by simply boosting the downstream-hosted innate immune response. Additionally, the reduced replication of RVFV in the *stat1b*^−/−^ larvae could be played in hand by a lower number of cells of the myeloid lineage, as indicated by low levels of *spi*, *mpx*, and *mpeg* markers. Since myeloid cells were present in reduced numbers, RVFV might have fewer opportunities to hide from the host innate immune response, which resulted in limited viral replication and more efficient infection control. While the levels of myeloid cell markers were also lower in the *stat1a*^−/−^ larvae, the virus could replicate in an unhindered manner due to the deficient host innate immune response.

Here, we show that zebrafish larvae are an excellent in vivo model to study RVFV replication, dissemination, and host innate immune response. Their versatility and ease of use make it a well-suited tool for high biosafety environments. Our results reveal that RVFV-induced blindness likely occurs due to direct damage to the eye and peripheral neurons, rather than to the central nervous system, and that the virus does so in a *stat1*-dependent manner.

This new RVFV model could be used to further dissect the interplays between virus and host and help to confirm the proposed association between innate immune gene polymorphisms and RVFV-associated clinical outcomes [61]. Also, the intricate innate immunity mechanisms activated to combat the RVFV infection that seem to directly influence which organs are affected can now be explored, thereby contributing to a better understanding of the pathogenesis of RVRV infection and to the discovery of efficacious therapies.

## Figures and Tables

**Table 2:** Antibodies used in whole mount immunohistochemistry.

Antigen	Host	Dilution	Source
GFP	Rabbit	1:1000	Chromotec (AB_2749857)
Red fluorescent protein (RFP)	Mouse	1:1000	Chromotec (AB_2631395)
Anti-Rabbit 488	Goat	1:500	Invitrogen (A-11008)
Anti-Mouse 594	Goat	1:500	Invitrogen (A-11032)

## Data Availability

The original contributions presented in this study are included in the article/Supplementary Material. Further inquiries can be directed to the corresponding author.

## Supplementary Materials

The following supporting information can be downloaded at: https://www.mdpi.com/article/10.3390/v17010087/s1, Figure S1: Input determination of RVFV35/74 and RVFVeGFP in zebrafish larvae.; Figure S2: Survival curves of RVFV infected zebrafish larvae.; Figure S3: Images of RVFVeGFP infected larvae where the infection has spread only limited beyond the optic tectum. Figure S4: Images of uninfected larvae: Table S1: Numbers of harvested and scored larvae per dpi of both RVFV_eGFP_ and PBS infected larvae; Table S2: Totals of GFP positivity rate per anatomical structure of the RVFV_eGFP_ and PBS injected larvae from six independent experiments. Video S1: Z-stack compilation of RVFVeGFP infected zebrafish larva.
