# Supplementary material for: The Dissemination of Rift Valley Fever Virus to the Eye and Sensory Neurons of Zebrafish Larvae Is Stat1-Dependent
Source: Viruses. 2025 Jan 11;17(1):87. doi: 10.3390/v17010087 (PMC11768566; doi:10.3390/v17010087)
Supplement: Supplementary file 1 [file viruses-17-00087-s001.zip › Supplementary figures.pdf]

Figure S1

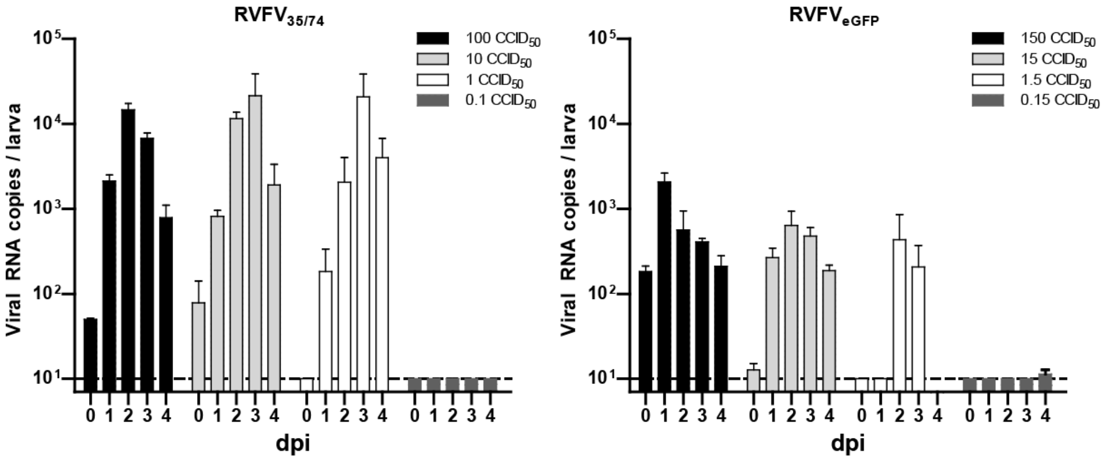

Figure S1 – Input determination of $RVFV_{35/74}$ and $RVFV_{eGFP}$ in zebrafish larvae. Viral RNA copies of $RVFV_{35/74}$ per zebrafish larva. Mean ± standard error of the mean (s.e.m.) of 2-4 pools of 10 larvae from 4 independent experiments. dpi = days post infection; RVFV = Rift Valley fever virus.

Figure S2

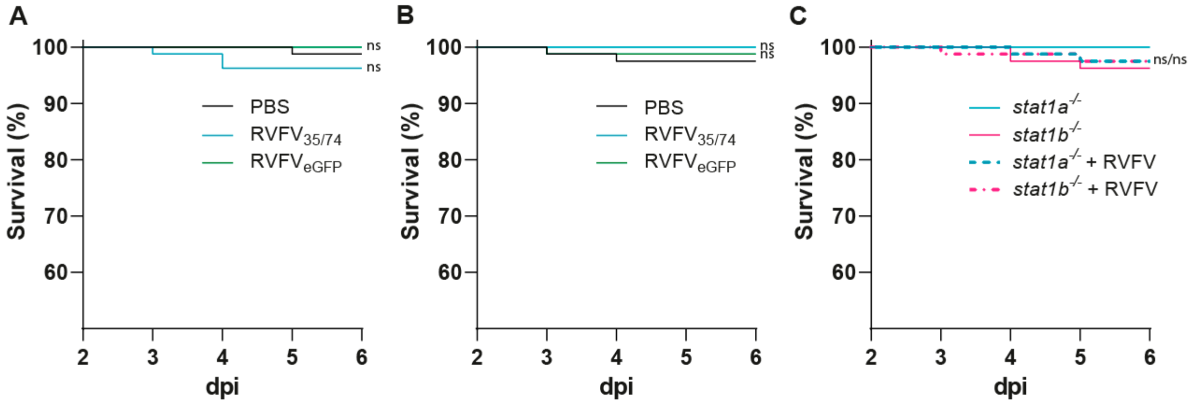

Figure S2 - Survival curves of RVFV infected zebrafish larvae. **A)** Survival of the larvae injected with $RVFV_{35\text{-}74}$, $RVFV_{eGFP}$ or PBS (uninfected). No significant larval death can be observed over the entire duration of the experiment. **B)** Survival of larvae infected with $RVFV_{35\text{-}74}$, $RVFV_{eGFP}$ or PBS (uninfected) while treated with ruxolitinib (continued immersion in Danieau's solution supplemented with 25 µM ruxolitinib). No significant larval death can be observed over the entire duration of the experiment. **C)** Survival of *stat1a*$^{-/-}$ and *stat1b*$^{-/-}$ larvae injected with $RVFV_{35\text{-}74}$ or PBS (uninfected). No significant larval death can be observed over the entire duration of the experiment. Statistical analysis between different groups was performed using Log-rank (Mantel-Cox) test where $p<0.05$ is considered significant. Data pooled from four independent experiments, total included numbers per group: n = 80. dpi = days post infection; RVFV = Rift Valley fever virus; ns = not significant

Figure S3

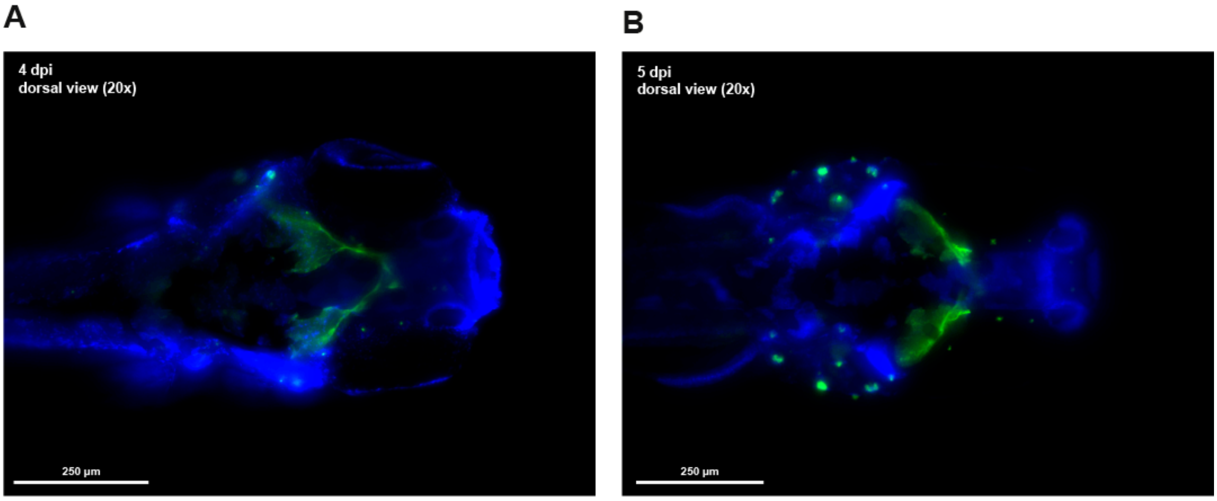

Figure S3 – Images of RVFV$_{eGFP}$ infected larvae where the infection has spread only limited beyond the optic tectum. Whole mount immunohistochemistry fluorescence images of RVFV$_{eGFP}$ infected zebrafish larvae at 20x magnification, using anti-GFP primary antibody and Hoechst 33342. Images were not 3D-deconvoluted. **A)** RVFV$_{eGFP}$ infected zebrafish larva at 4 dpi showing only infection of the optic tectum and the most proximal neuromasts. **B)** RVFV$_{eGFP}$ infected zebrafish larva at 5 dpi showing infection in the optic tectum and proximal neuromasts.

Figure S4

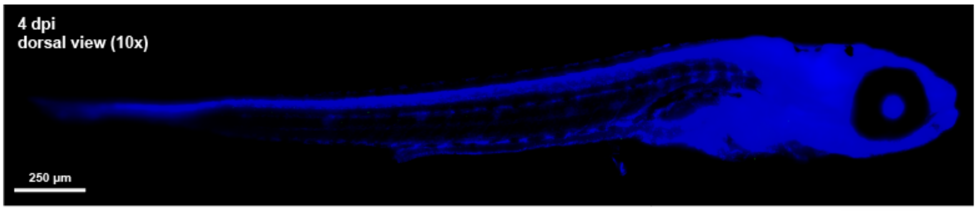

Figure S4 – Images of uninfected larvae. Whole mount immunohistochemistry fluorescence images of uninfected PBS injected zebrafish larvae at 10x magnification, using anti-GFP primary antibody and Hoechst 33342. Image is not 3D-deconvoluted.

Table S1 & S2

Table S1: Numbers of harvested and scored larvae per dpi of both $RVFV_{eGFP}$ and PBS infected larvae. A total of 180 PBS controls over six independent experiments were harvested for analyzes. (Example: '10/5' = 10 $RVFV_{eGFP}$ injected larvae were scored / 5 PBS injected larvae were scored.)

AB larvae harvest numbers	*$RVFV_{eGFP}$ injected larvae / PBS injected larvae*						
	**0 dpi (72 hpf)**	**1 dpi (96 hpf)**	**2 dpi (120 hpf)**	**3 dpi (144 hpf)**	**4 dpi (168 hpf)**	**5 dpi (192 hpf)**	**6 dpi (216 hpf)**
Exp 1	10/5	20/5	20/5	20/5	20/5	-	-
Exp 2	10/5	20/5	20/5	20/5	20/5	-	-
Exp 3	10/5	20/5	20/5	20/5	20/5	-	-
Exp 4	5/5	5/5	5/5	5/5	5/5	5/5	5/5
Exp 5	5/5	5/5	5/5	5/5	5/5	5/5	5/5
Exp 6	5/5	5/5	5/5	5/5	5/5	5/5	5/5
Total	45/30	75/30	75/30	75/30	75/30	15/15	15/15

Table S2: Totals of GFP positivity rate per anatomical structure of the $RVFV_{eGFP}$ and PBS injected larvae from six independent experiments. The site of injection (or surrounding structures) were so bright green that individual organs/tissues could not be distinct from 3 dpi onward and as such was not quantified beyond that timepoint. Out of the 180 PBS injected larvae that were scored under the microscope none showed GFP signal. n=6. nq = not quantifiable.

Infection positivity scoring	*$RVFV_{eGFP}$ injected larvae / PBS injected larvae*						
	**0 dpi**	**1 dpi**	**2 dpi**	**3 dpi**	**4 dpi**	**5 dpi**	**6 dpi**
Site of injection	0/0*	44/0*	52/0*	nq/0	nq/0	nq/0	nq/0
Liver	0/0	11/0	37/0	33/0	45/0	6/0	8/0
vasculature	0/0	0/0	0/0	0/0	0/0	1/0	0/0
neuromasts	0/0	0/0	8/0	20/0	21/0	5/0	4/0
Optic tectum	0/0	0/0	8/0	20/0	22/0	5/0	5/0
Others (retina, macula)	0/0	0/0	4/0	12/0	12/0	3/0	4/0

* n=3
